# Chloroplast Protein Degradation in Senescing Leaves: Proteases and Lytic Compartments

**DOI:** 10.3389/fpls.2019.00747

**Published:** 2019-06-19

**Authors:** Agustina Buet, M. Lorenza Costa, Dana E. Martínez, Juan J. Guiamet

**Affiliations:** Instituto de Fisiología Vegetal (INFIVE, CONICET-UNLP), La Plata, Argentina

**Keywords:** leaf senescence, chloroplast protein degradation, protease, SAG12, vacuole, senescence-associated vacuoles

## Abstract

Leaf senescence is characterized by massive degradation of chloroplast proteins, yet the protease(s) involved is(are) not completely known. Increased expression and/or activities of serine, cysteine, aspartic, and metalloproteases were detected in senescing leaves, but these studies have not provided information on the identities of the proteases responsible for chloroplast protein breakdown. Silencing some senescence-associated proteases has delayed progression of senescence symptoms, yet it is still unclear if these proteases are directly involved in chloroplast protein breakdown. At least four cellular pathways involved in the traffic of chloroplast proteins for degradation outside the chloroplast have been described (i.e., “Rubisco-containing bodies,” “senescence-associated vacuoles,” “ATI1-plastid associated bodies,” and “CV-containing vesicles”), which differ in their dependence on the autophagic machinery, and the identity of the proteins transported and/or degraded. Finding out the proteases involved in, for example, the degradation of Rubisco, may require piling up mutations in several senescence-associated proteases. Alternatively, targeting a proteinaceous protein inhibitor to chloroplasts may allow the inhibitor to reach “Rubisco-containing bodies,” “senescence-associated vacuoles,” “ATI1-plastid associated bodies,” and “CV-containing vesicles” in essentially the way as chloroplast-targeted fluorescent proteins re-localize to these vesicular structures. This might help to reduce proteolytic activity, thereby reducing or slowing down plastid protein degradation during senescence.

## Introduction

The final phase of leaf development, senescence, is a process that precedes cell death, and it is characterized by chloroplast breakdown, with degradation and loss of chloroplast proteins, nucleic acids, pigments, lipids, and polysaccharides (e.g., starch). This deterioration is so extensive that chloroplasts eventually lose all or most of their photosynthetic capacity. Therefore, delaying senescence and thereby extending canopy photosynthesis might be viewed as a plausible goal of crop breeding to increase canopy C gain and grain yield. However, since chloroplasts contain up to 70% of leaf N, the breakdown of leaf proteins and redistribution of released amino acids to other parts of the plant (for example, immature growing seeds) can have a positive impact on the nitrogen use efficiency of crops (Gregersen et al., 2008). Depending on crop species, growth conditions, or the eventual use of the harvested product (e.g., food with high nutritional value or biofuel), it might be desirable to delay or accelerate senescence in order to increase either C gain or N use efficiency. To manipulate leaf N remobilization, we need a fine understanding of the mechanisms responsible for the regulation and execution of leaf chloroplast protein breakdown, and particularly of the cellular pathways involved and the proteases with a crucial role in this process. This review will focus on proteases associated with senescence and their possible role(s) in chloroplast protein breakdown, as the basis for both decreased photosynthetic capacity of senescing leaves and N redistribution. Since different cellular compartments apparently involved in chloroplast protein breakdown have emerged during the last 15 years, chloroplast protein trafficking for degradation and the cellular compartments possibly involved in the execution of protein degradation will also be discussed.

## Increased Degradation, Not Reduced Rates of Synthesis, Drives the Decrease of Chloroplast Protein Levels

The steady-state levels of most photosynthetic proteins decrease markedly during senescence (Krupinska and Humbeck, 2004). Although this might be due to a combination of decreased synthesis plus enhanced degradation rates, the evidence shows clearly that rates of protein synthesis become negligible after complete leaf expansion (Makino et al., 1984; Mae, 2004), and expression of photosynthetic genes typically declines sharply in fully expanded, yet non-senescent leaves (e.g., Krupinska and Humbeck, 2004; Breeze et al., 2011). Chloroplast protein levels are mostly regulated by rates of degradation during senescence (Lamattina et al., 1985; Mae, 2004). An exception is the D1 protein, which is constantly subjected to proteolysis and resynthesis as part of a photodamage repair cycle (Aro et al., 1993; Guiamét et al., 2002); but in spite of its potential impact on photosynthetic rates, breakdown of D1 may not make a large contribution to N redistribution.

## Expression and Activity of Proteases During Senescence

Given the crucial role of protein degradation in the decline of photosynthetic protein levels, detection of proteases whose activity or expression increases during leaf senescence might contribute to identify putative candidate genes to manipulate senescence. Transcriptomic studies have consistently shown, across a range of different plant species, that some of the genes up-regulated during senescence (“senescence-associated genes,” SAGs) are proteases (e.g., Parrott et al., 2007; Breeze et al., 2011; Sekhon et al., 2012; Costa et al., 2013; Zhang et al., 2014). Most proteases associated with senescence are serine and cysteine (Cys) proteases, but some are aspartic proteases and metalloproteases (Roberts et al., 2012; Díaz-Mendoza et al., 2014). Also vacuolar processing enzymes (VPEs) are up-regulated at the mRNA level during senescence (Kinoshita et al., 1999).

The chloroplast seems a logical place to harbor proteases involved in the degradation of photosynthetic proteins. The major chloroplast protease families (Clp, FtsH, DegP) display mostly constitutive expression and seem to be involved in protein quality control and maintenance of homeostasis rather than in massive protein degradation (van Wijk, 2015), although some members of these families can be up-regulated during leaf senescence (Costa et al., 2013). The FtsH6 metalloprotease has been linked to senescence as it was first shown to degrade Lhcb3 *in vitro* (Zelisko et al., 2005), but this result could not be confirmed *in vivo* (Wagner et al., 2011). Another metalloprotease, M58, was shown to localize to plastoglobules (i.e., lipid droplets that accumulate within plastids during senescence), although its function remains unknown (Lundquist et al., 2012).

Several recent studies using *in vitro* protease assays in the presence of class-specific inhibitors, or class-specific substrates, have shown that cysteine proteases are the most active in senescing leaves (i.e, leaves undergoing rapid protein degradation) and that their expression and activity increase substantially during senescence (e.g., Beyenne et al., 2006; Martínez et al., 2007; Carrión et al., 2013; Poret et al., 2016). Most cysteine proteases associated with senescence are located to the central vacuole (Martínez et al., 2007), or other lytic compartments (Costa et al., 2013). Among Cys proteases, cathepsins are highly expressed and active during senescence in *Arabidopsis* (McLellan et al., 2009) and barley (Velasco-Arroyo et al., 2016). RD21 and aleurain are also cysteine proteases associated with senescence in various different species (van der Hoorn et al., 2004; Poret et al., 2016), and they comprise the largest cysteine protease activity of senescing leaves (Pružinská et al., 2017). Several of the proteases associated to senescence are also expressed and/or active in other developmental processes or under different environmental conditions (Martínez et al., 2007). For example, RD21 was initially discovered as a drought-inducible gene (Koizumi et al., 1993). The Cys protease SAG12 was discovered by Lohman et al. (1994) in a search for genes with increased expression during senescence. SAG12 is classified into the cathepsin L-like family, subgroup A (Díaz-Mendoza et al., 2014). Unlike other SAGs, which show a basal level of expression in mature leaves and up-regulation during senescence, SAG12 transcripts are almost undetectable in mature leaves, and SAG12 is expressed exclusively during senescence (Lohman et al., 1994; Grbic, 2002, 2003; Gombert et al., 2006). The senescence-specific responsive element in the SAG12 promoter is located between −603 and −571 bp in the 5′ region (Noh and Amasino, 1999). SAG12 is also expressed in flowers, more specifically in the corolla limb and corolla abscission zone, in anthers and pistils of pollinated flowers (Grbic, 2002), in unfertilized pistils (Carbonell-Bejerano et al., 2011), and in *Arabidopsis* roots (James et al., 2019). The regulated induction of SAG12 has been exploited to use the SAG12 promoter to drive the senescence-associated expression of IPT, the key gene in cytokinin biosynthesis, to delay senescence in an autoregulated manner (Gan and Amasino, 1995). This approach has been used successfully in various species (e.g., lettuce, McCabe et al., 2001, wheat, Sýkorová et al., 2008, and rice, Liu et al., 2010). Likewise, Liu et al. (2010) described a cysteine protease of rice named SAG39, homologous to AtSAG12, whose expression increases in leaves, roots, culms, and flowers during natural senescence. Vacuolar processing enzymes (VPEs) are a class of Cys proteases likely involved in activation of vacuolar proteases through proteolytic cleavage of inhibitory peptides (Kinoshita et al., 1999). Although VPE mRNAs increase in abundance during senescence, VPE activity may actually decrease in *Arabidopsis* (Pružinská et al., 2017), while in *Brassica napus*, VPE activity increases during senescence to a similar extent in genotypes with different rates of protein breakdown (Poret et al., 2019), casting doubts on VPE involvement in senescence-associated protease activation and protein breakdown.

To strengthen the association of protease activity and/or expression with senescence progression, the rate of senescence can be manipulated through the use of hormonal treatments. Typically, cytokinins delay senescence, while ethylene, abscisic acid (ABA), and salicylic acid (SA) accelerate it (Jibran et al., 2013). Often, hormonal treatments that delay senescence reduce the activity of senescence-associated proteases (e.g., Fukayama et al., 2010; Carrión et al., 2013). Conversely, treatment with ethylene (or its precursor, 1-aminocyclopropane-1-carboxylic-acid), abscisic acid, or salicylic acid increases protease activity/expression concomitantly with accelerated degradation of the photosynthetic machinery (e.g., Chen et al., 2006; Fukayama et al., 2010; Liu et al., 2010; Poret et al., 2017).

## Knockouts, Silenced or Overexpressing Lines, and *in Vivo* Pharmacological Inhibition of Proteases

Increased expression or activity of a protease suggests a role during senescence, but a stronger proof may come from the functional analysis where expression is silenced or knocked down, or where pharmacological approaches are used to decrease protease activity *in vivo*.

In some cases, functional analyses of senescence-associated proteases have shown clearly their involvement in processes other than bulk chloroplast protein degradation and N remobilization. For example, the apoplast-localized subtilisin protease AtSASP (AtSBT1.4) is highly up-regulated at the transcript and activity levels during senescence (Martínez and Guiamet, 2014; Martinez et al., 2015). Knockout *sasp1* plants show no obvious phenotype at juvenile stages, but at the reproductive stage *sasp1* plants develop more branches and siliques, with no significant alteration of leaf senescence (Martinez et al., 2015). Knockout *sasp1* plants are also more sensitive to abscisic acid (ABA) and, therefore, more tolerant to drought (Wang et al., 2018). SASP apparently controls ABA sensitivity by increasing the degradation of Open Stomata 1 (Wang et al., 2018), a positive regulator of ABA-mediated stomatal closure (Acharya et al., 2013). Thus, although SASP is clearly a senescence-associated protease, it is not part of a bulk chloroplast protein degradation and N remobilization pathway, and appears to have a role in diverse regulatory pathways, likely attenuating responses to ABA. It is possible that other senescence-associated proteases function in developmental regulation rather than in direct bulk chloroplast protein breakdown. Expression of the chloroplast-located aspartic protease CND41 increases during senescence, and CND41 degrades partially denatured Rubisco *in vitro* (Kato et al., 2004). CND41 antisense and overexpressing lines of tobacco display retarded and enhanced senescence, respectively, during vegetative growth before anthesis (Kato et al., 2004, 2005), which is in line with its putative involvement in Rubisco degradation. However, the antisense lines where Rubisco degradation is delayed are also deficient in gibberellins and stunted in growth (Nakano et al., 2003); since senescence of a leaf often depends on the correlative influence of younger leaves, slower growth complicates the interpretation of CDN41 results. Testing the impact of protease inactivation in different senescence scenarios (e.g, *in planta* during natural senescence and in excised leaves where effects of N redistribution on whole plant physiology are avoided) may help to ascribe a role for a given protease in chloroplast protein breakdown. These examples illustrate the complexities in assigning roles in chloroplast breakdown to proteases with expression temporally associated with senescence.

The involvement of Cys proteases in Rubisco degradation has been probed using a pharmacological approach with specific inhibitors. Although in recent years, a variety of protease inhibitors have been modified to label proteases and facilitate their detection and isolation, few of these chemicals have been tried *in vivo*, i.e., to block protease activity and examine changes in the stability of specific proteins. E-64 has been broadly used as a diagnostic inhibitor of cysteine proteases *in vitro*, and a few works have extended its application to living tissues. Thoenen et al. (2007) floated wheat leaf segments on 0.1 mM E-64 and found slower degradation of Rubisco large subunit and Rubisco activase, either in leaf segments maintained in darkness for 4 days or after incubation in 25 mM KCl for 7 days under dim light (25 μmol m^−2^ s^−1^ of photosynthetic photon flux density). In a similar approach, tobacco leaf disks pre-treated with ethephon to accelerate senescence were floated on E-64 in darkness for 2 days; E-64 significantly reduced Rubisco loss under these conditions (Carrión et al., 2013). Inhibition of Rubisco degradation closely mirrored the inhibition of cysteine protease activity in these leaves, as determined by microscopic observation of cells stained with a Cys protease fluorescent substrate probe. These studies clearly point to Cys proteases as important in the degradation of Rubisco, but they provide no information on the identity of the protease(s) involved or their specific function.

Reverse genetics approaches provide evidence for the participation of Cys proteases in leaf senescence. Seeβ is a senescence-associated gene coding for a legumain-type Cys protease of maize (Donnison et al., 2007). A screening of a maize Mutator (Mu) population identified a line with a Mu insertion in Seeβ. Under two N supply regimes, the mutant plants had several characteristics consistent with a role for Seeβ in leaf senescence, including slightly higher and more persistent leaf N contents, which might indicate slower and/or incomplete protein degradation and less N export. Whether the mutation impairs the breakdown of specific leaf proteins was not reported.

Three different genes code for the Cys protease Cathepsin B (CathB) in *Arabidopsis*, and they redundantly function in the development of programmed cell death in response to pathogens. CathB genes are also up-regulated during developmental leaf senescence (McLellan et al., 2009). Interestingly, senescence (monitored as loss of chlorophyll) is delayed in a triple CathB mutant. CathB may act upstream of other senescence-associated proteases regulating their expression, since, for example, SAG12 expression is much reduced in the triple CathB mutant. Although this functional analysis clearly places cathepsins in a pathway regulating senescence, protein targets of CathB are still unknown. A functional analysis of several Papain-like Cysteine proteases (e.g., RD21, SAG12, CTB3, aleurain) failed to detect differences in chlorophyll content between wild type and mutant leaves incubated in darkness for 7 days (Pružinská et al., 2017). However, by counting the number of green vs. yellow leaves per plant, a delay in plant senescence was detected for lines knock-out (KO) for aleurain. Both studies (McLellan et al., 2009; Pružinská et al., 2017) pinpoint proteases with a possible role in the regulation of senescence, but reliance on chlorophyll as the only senescence parameter does not allow to draw conclusions on the role of these proteases in chloroplast protein breakdown. As shown by a number of studies, degradation of pigments and that of proteins appear to be two rather independent processes during senescence of leaves (Thomas and Howarth, 2000; Guiamét et al., 2002).

Cys proteases associated with senescence were studied in barley leaves, and one of them, HvPAP1, was silenced and overexpressed (Velasco-Arroyo et al., 2016). Overexpression of HvPAP1 accelerated the development of senescence symptoms (yellowing), whereas the knockdown lines showed delayed senescence during the reproductive phase. When challenged by stress conditions (i.e., continuous darkness or N deprivation), the decrease in leaf protein content was less marked in the knockdown lines, as expected if HvPAP1 were directly involved in protein remobilization. Inactivation of HvPAP1 also caused some changes in the levels of active/inactive HvPAP16, which uncovers another possible function for HvPAP1, i.e., maturation of other cysteine proteases. Barley Cys proteases (e.g., HvPAP1 and HvPAP19) are up-regulated in response to water deficit, and knockdowns for HvPAP1 and HvPAP19 reduce leaf protein degradation under stress conditions (Gomez-Sanchez et al., 2019).

Otegui et al. (2005) found no visual differences in senescence progression between a wild type and a SAG12 knockout line, but protein degradation was not assessed in that study. James et al. (2018) reported lower harvest index and N harvest index in plants of *Arabidopsis* KO for SAG12 growing under a low nitrogen supply, indicating impaired N redistribution in this mutant. It is noteworthy that the expression of an aspartic protease (the product of At5g10760), and overall activity of the aspartic protease class, increased in the SAG12 KO, possibly reflecting the occurrence of functional protease redundancy during senescence of leaves. Interestingly, inactivation of SAG12 also reduced N redistribution from roots under low nitrogen nutrition (James et al., 2019), suggesting a similar function for SAG12 in such functionally different organs as leaves and roots.

The function of SAG12 in other species is much less clear. In rice, down-regulation of either of two putative orthologs of SAG12 (i.e., OsSAG12-1 and OsSAG12-2) accelerates cell death in response to biotic and abiotic stresses (Singh et al., 2013, 2016). Whether this is due to a different role for SAG12 in different species (e.g., monocots vs. dicots) or to difficulties in uncovering orthologs with SAG12 functions based only on sequence similarities deserves further studies. For example, in order to identify an AtSAG12 ortholog gene in maize (*Zea mays*, B73 inbred line), a phylogenetic tree was constructed including AtSAG12 and ortholog genes reported from other species (i.e., *Brassica napus*, *Nicotiana tabaccum*, *Oryza sativa*, and *Ipomoea batatas*). The resulting tree showed more than 10 maize genes closely related to rice OsSAG39, a putative SAG12 ortholog. All the maize genes analyzed show high percent identity with OsSAG39. However, a search of the expression pattern of five of these genes employing Maize eFP Browser^1^ showed that AC225716.2_FG003, AC225716.2_FG006, GRMZM2G028862, GRMZM2G137690, and GRMZM2G165086 show high expression levels in germinating seeds but low expression in senescing leaves, while GRMZM2G095628 shows high expression in germinating seeds and senescing leaves, among other organs (Sekhon et al., 2011). Pinpointing SAG12 orthologs in species other than *Arabidopsis* may require in-depth studies.

Although functional analysis is a robust approach to probe the involvement of specific proteases in chloroplast protein breakdown, pleiotropic effects of proteases may complicate the interpretation of such studies. Reduced growth or development of mutant lines, mentioned before for CND41 (Nakano et al., 2003), or increased cuticle thickness and altered stomatal dimensions in knockdown HvPAP1 and HvPP19 barley lines (Gomez-Sanchez et al., 2019) are good examples of this problem.

## Endogenous Protease Inhibitors

In addition to changes in protease activity, increased protein degradation during senescence might be related to decreased abundance of endogenous protease inhibitors (Etienne et al., 2007; Martínez et al., 2012; Díaz-Mendoza et al., 2014). Serpins (Fluhr et al., 2012) may be key inhibitors of senescence-associated proteases, such as RD21 (Lampl et al., 2010) and also of metacaspases involved in programmed cell death (Cohen et al., 2019). Other protease inhibitors likely involved in senescence include the Kunitz-type trypsin inhibitor WSCP, which interacts and inhibits mature RD21 and participates in the control of programmed cell death in the transmitting tract of *Arabidopsis* flowers (Boex-Fontvieille et al., 2015). Given the prevalence of C1A cysteine proteases among senescence-associated proteases, phytocystatins, their specific proteinaceous inhibitors, are of special interest (Díaz-Mendoza et al., 2014). Phytocystatins are encoded by small gene families comprised of seven members in *Arabidopsis* and 12 in rice (Martínez et al., 2005). In some species, levels or expression of cystatins decrease in senescing leaves, presumably allowing for increases in cysteine protease activity (Tajima et al., 2011), whereas in barley cystatin, expression increases under senescence-inducing conditions, e.g., protracted darkness (Díaz-Mendoza et al., 2014). Overexpression of oryzacystatin (OC) in soybean leads to increased branching and delayed senescence; however, both in soybean and *Arabidopsis*, OC-overexpressing lines are slightly delayed in growth (Quain et al., 2014), which might partly explain delayed senescence symptoms. Similar growth effects of overexpression of OC were seen in tobacco (Prins et al., 2008), where cystatin targeted to the cytosol also resulted in delayed degradation of Rubisco and Rubisco activase. It is interesting to note that in this study, OC was localized to the cytosol, whereas Rubisco was located in chloroplasts and in defined non-chloroplastic vesicular structures (i.e., “Rubisco vesicular bodies”). The mechanism for delayed degradation of Rubisco in OC-expressing lines remains to be studied.

## Cellular Pathways Involved in Chloroplast Protein Breakdown

**Figure 1:**
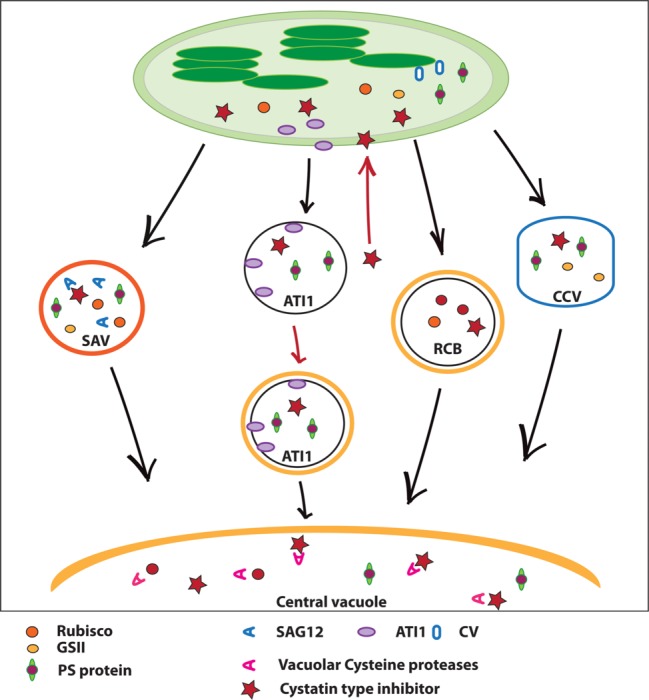
A schematic representation of cellular pathways delivering chloroplast proteins to lytic compartments and a proposed strategy to inhibit plastid protein degradation in crop species. Chloroplast proteins traffic to “senescence-associated vacuoles” (SAVs) and/or to the central vacuole *via* “ATI1-plastid associated bodies” (ATI1-PS), “Rubisco-containing bodies” (RCBs), or “CV-containing vesicles” (CCVs). Each of these vesicles apparently transports specific proteins (Rubisco for SAVs and RCBs, certain thylakoid proteins for ATI1-PS and CCV), which may be degraded by cysteine proteases either in SAVs or the central vacuole. SAVs might also be internalized into the central vacuole, although this is hypothetical. A proposed strategy to reduce/slow down chloroplast protein degradation during senescence in crop plants is the use of targeted protease inhibitors. A proteinaceous protease inhibitor (e.g., a phytocystatin, or a Kunitz-type inhibitor such as WSCP, represented by a red star in the cytosol) fused to a chloroplast transit peptide is expressed under control of a senescence-induced promoter. The chloroplast-located inhibitor would then be redirected to RCBs, ATI1-PS, CCV, and/or SAVs by the same mechanism which directs chloroplast-targeted fluorescent proteins to these vesicular structures. Once in the central vacuole or in SAVs, the inhibitor binds proteases, thus reducing proteolytic activity and preserving protein levels.

In recent years, a number of papers have described protein-trafficking pathways delivering chloroplast proteins to lytic compartments, e.g., the central vacuole or “senescence-associated vacuoles” (Xie et al., 2015). These pathways and lytic compartments might play an important role in the degradation of some of the most abundant chloroplast proteins, e.g., Rubisco (Figure 1).

Ishida and co-workers discovered “Rubisco-Containing Bodies” (RCB) in the cytosol of senescing wheat and *Arabidopsis* leaves (Chiba et al., 2003; Ishida et al., 2008). RCBs are spherical vesicles, about 1 μm in diameter, surrounded by a double membrane, and contain Rubisco and glutamine synthetase II. RCBs are eventually internalized into the central vacuole, where their cargo chloroplast proteins are degraded. Both, formation and vacuolar internalization of RCBs depend on the autophagic pathway (Ishida et al., 2008; Wada et al., 2009), implying the involvement of autophagy in chloroplast protein breakdown. Thus, it is intriguing that the phenotype of autophagy mutants includes accelerated senescence and premature loss of chloroplast proteins (e.g., Hanaoka et al., 2002; Thompson et al., 2005; Guiboileau et al., 2013). Accelerated senescence is a typical phenotype for autophagy mutants, and this includes *Arabidopsis* mutants in the autophagy genes ATG7 (Doelling et al., 2002), ATG4 (Hanaoka et al., 2002), ATG5 (Thompson et al., 2005), ATG10 (Phillips et al., 2008), and maize mutant for the ATG12 gene (Li et al., 2015). Accelerated senescence in autophagy mutants involves a salicylic acid-dependent pathway (Yoshimoto et al., 2009), which is reminiscent of SA regulation of normal leaf senescence (Morris et al., 2000). Lack of autophagy seems to be compensated for by increased expression of senescence-associated cysteine proteases, e.g., SAG12 (Havé et al., 2018). This implies some degree of functional redundancy, and exacerbation of separate senescence-associated proteolytic pathways when canonical autophagy is blocked.

Using the senescence-associated protease SAG12 fused to GFP, Otegui et al. (2005) localized SAG12 to small, acidic, proteolytically active “senescence-associated vacuoles” (SAVs), which are completely absent from mature, non-senescing leaves but appear in substantial numbers during senescence. SAVs are bound by a single membrane, and their diameter ranges from 500 to 800 nm. SAVs were also shown to contain stromal (e.g., Rubisco and Glutamine synthetase II) and thylakoid (e.g., PsaA and Lhcas) proteins, indicating trafficking between the plastid and SAVs in senescing leaves (Martínez et al., 2008; Gomez et al., 2019). Remarkably, SAVs are devoid of PSII components (Gomez et al., 2019). The involvement of SAVs in chloroplast protein degradation was indicated by *in vitro* autodigestion of chloroplast proteins contained within isolated SAVs (Martínez et al., 2008; Gomez et al., 2019), and by *in vivo* experiments where incubation of leaf disks with the cysteine protease inhibitor E-64 completely abolished the protease activity of SAVs and concomitantly reduced Rubisco degradation (Carrión et al., 2013). Since SAVs contain SAG12, the recent finding that leaf N redistribution is impaired in a SAG12-KO line (James et al., 2018) lends support to the idea that SAVs participate in chloroplast protein breakdown. Unlike RCBs, formation of SAVs does not require functional autophagy (Otegui et al., 2005). Their proteolytic activity distinguishes SAVs from all other vesicular pathways associated to senescence described so far.

Two other chloroplast protein-trafficking vesicles are well characterized. ATG8-Interacting Protein 1 (ATI1) participates in vesicular trafficking to the central vacuole from the ER (Honig et al., 2012) and from chloroplasts (Michaeli et al., 2014). ATI1 can be detected within plastids, and also in the cytosol, associated with vesicles (ATI1-plastid associated bodies, ATI1-PS) containing chloroplast-targeted GFP. Formation of ATI1-PS and their release from plastids into the cytosol do not require the autophagic machinery, whereas their internalization in the central vacuole requires the operation of functional autophagy, and it is therefore blocked in ATG5 knockout mutants (Michaeli et al., 2014). ATI1 interacts with several chloroplast proteins, most of them located to the thylakoids, and knocking out ATI1 reduces the degradation of chloroplastic Cys peroxiredoxin, suggesting that Cys peroxiredoxin is broken down through this pathway. Confocal images also show a fluorescent signal corresponding to chlorophyll in ATI1-PS.

The above mentioned pathways require relocation of chloroplast proteins outside the plastid. Recently, the CV (chloroplast vesiculation) gene was identified, and shown to increase in expression during natural and abiotic stress-induced senescence (Wang and Blumwald, 2014). The CV protein localizes to plastids, where it interacts with a number of chloroplast proteins, mainly thylakoid components. CV causes the formation of intra-plastidic vesicles (CV-containing vesicles, CCV) that eventually bud off the plastid and move to the central vacuole, carrying stromal and thylakoid proteins. Overexpression of CV under control of an inducible promoter accelerates senescence, reducing the abundance of PSI and PSII proteins, as well as Glutamine Synthetase II. CCVs are apparently devoid of SAG12, and the CCV pathway remains active in ATG5 knockout mutants, suggesting that this pathway is independent of autophagy.

## Conclusions/Outlook

It is quite possible that chloroplast protein breakdown in senescing leaves is achieved by the coordinated operation of at least several proteases, which might also operate in different lytic compartments, e.g., SAG12 and possibly other proteases within SAVs, or proteases in the central vacuole (e.g., RD21, aleurain, etc.) acting on chloroplast proteins carried there by Rubisco-containing bodies, ATI-PS Bodies, and/or CCVs (Figure 1; Costa et al., 2013; Avila-Ospina et al., 2014; Ishida et al., 2014; Michaeli et al., 2014; Wang and Blumwald, 2014; Xie et al., 2015). Such redundancy might explain the relatively small effects of, for example, knocking out one particular protease (e.g., Pružinská et al., 2017). Similarly, the existence of several apparently independent vesicular pathways for chloroplast protein degradation outside the plastid is intriguing. It might be argued that each of these vesicles carries a specific set of chloroplast proteins, affording the cell with the flexibility needed to independently degrade stromal and thylakoid proteins, or PSI vs. PSII components, with different time-courses depending on, for example, environmental conditions. Indeed, pharmacological inhibition of cysteine protease activity in SAVs delayed Rubisco degradation *in vivo* (Carrión et al., 2013), inactivation of ATI1 preserved Cys peroxiredoxin (Michaeli et al., 2014) and overexpression of CV under control of an inducible promoter-accelerated degradation of the thylakoid proteins PsaB, PsbA, and PsbO1 (Wang and Blumwald, 2014). Protein-protein interaction assays also show that ATI1 and CV possibly interact with defined sets of chloroplast proteins (Michaeli et al., 2014; Wang and Blumwald, 2014). While this evidence supports the idea that each pathway might be involved in the breakdown of specific plastid proteins, the fact that all these vesicles are loaded with chloroplast-targeted, fluorescent reporter proteins argues against specificity in their cargo. Thus, whether each of these pathways is specifically involved in the transport and eventual degradation of particular sets of chloroplast proteins is not definitively established. Likewise, each of these structures has so far been probed independently with different fluorescent proteins or activity markers; therefore, examination of senescing cells with combinations of molecular markers for each of these vesicles (e.g., SAG12:GFP plus ATI1:mCherry) would help to establish whether they are truly independent structures or, in some cases, the same vesicles visualized with different tools. To some extent, this has been already done for CCVs (Wang and Blumwald, 2014), but the approach should be extended to the remaining pathways.

If, as presumed, there is redundancy in terms of the proteases and proteolytic pathways involved, manipulation of chloroplast protein degradation to prolong photosynthetic activity in crops might imply piling up several mutations (e.g., through CRISPR editing) in different proteases to decrease proteolytic activity significantly. An alternative approach might be to express protein inhibitors of cysteine proteases. Work by Thoenen et al. (2007) and Carrión et al. (2013) indicate that cysteine proteases are responsible for a significant part of the degradation of chloroplast proteins. Expressing phytocystatins, for example, might be effective in reducing protein degradation. As already shown for fluorescent proteins, cystatins targeted to plastids might traffic to the intended proteolytic compartments, SAVs, or to the central vacuole *via* RCBs, CCVs, or ATI1-PS (Figure 1). To minimize the risk of side-effects, such as slower growth as in the case of constitutive expression of oryzacystatin in the cytosol (Prins et al., 2008), a senescence induced promoter (e.g., pSAG12, Gan and Amasino, 1995) might be used to restrict cystatin expression to senescing leaves. This might represent a feasible approach to reduce cysteine protease activity and partially block protein degradation in senescing leaves of crop plants.

## Author Contributions

AB, MC, DM, and JG carried out the literature search and wrote the manuscript.

### Conflict of Interest Statement

The authors declare that the research was conducted in the absence of any commercial or financial relationships that could be construed as a potential conflict of interest.

## Footnotes

^1^http://bar.utoronto.ca/efp_maize/cgi-bin/efpWeb.cgi
